# Investigation of hearing loss in elderly vertigo and dizziness patients in the past 10 years

**DOI:** 10.3389/fnagi.2023.1225786

**Published:** 2023-09-15

**Authors:** Qian Wang, Aiting Chen, Mengdi Hong, Xingjian Liu, Yi Du, Ziming Wu, Wenbo Cheng, Fei Ji

**Affiliations:** ^1^Department of Otolaryngology-Head and Neck Surgery, The Medical Center of PLA General Hospital, Beijing, China; ^2^National Clinical Research Center for Otolaryngologic Diseases, Beijing, China; ^3^State Key Laboratory of Hearing Science, Ministry of Education, Beijing, China; ^4^Beijing Key Laboratory of Hearing Impairment Prevention and Treatment, Beijing, China; ^5^Academy of Medical Technology and Information Engineering, Zhejiang Chinese Medical University, Hangzhou, China

**Keywords:** hearing loss, vertigo, elderly, dizziness, pure tone audiometry, acoustic immittance measurement, auditory brainstem response (ABR)

## Abstract

**Background:**

Vertigo and hearing loss are both prevalent in the elderly. This study retrospectively analyzed hearing test results from elderly patients experiencing vertigo and dizziness at ENT outpatient over a 10-year period, in order to study the patterns of hearing loss in this patient population.

**Methods:**

Nine thousand three hundred eighty four patients over 50 years old underwent retrospective collection and screening of outpatient diagnosis, pure tone audiometry, acoustic immittance measurement (tympanogram) and auditory brainstem response (ABR) test. The patient's audiograms are divided into 7 subtypes according to a set of fixed criteria. Meanwhile, K-Means clustering analysis method was used to classify the audiogram.

**Results:**

The Jerger classification of tympanogram in elderly patients with vertigo and dizziness showed the majority falling under type A. The leading audiogram shapes were flat (27.81% in right ear and 26.89% in left ear), high-frequency gently sloping (25.97% in right ear and 27.34% in left ear), and high-frequency steeply sloping (21.60% in right ear and 22.53% in left ear). Meniere's disease (MD; 30.87%), benign recurrent vertigo (BRV; 19.07%), and benign paroxysmal positional vertigo (BPPV; 15.66%) were the most common etiologies in elderly vestibular diseases. We observed statistically significant differences in hearing thresholds among these vestibular diseases (*P* < 0.001). K-Means clustering analysis suggested that the optimal number of clusters was three, with sample sizes for the three clusters being 2,747, 2,413, and 4,139, respectively. The ANOVA statistical results of each characteristic value showed *P* < 0.001.

**Conclusion:**

The elderly patients often have mild to moderate hearing loss as a concomitant symptom with vertigo. Female patients have better hearing thresholds than males. The dominant audiometric shapes in this patient population were flat, high-frequency gently sloping, and high-frequency steeply sloping according to a set of fixed criteria. This study highlights the need for tailored strategies in managing hearing loss in elderly patients with vertigo and dizziness.

## 1. Introduction

Previous studies indicate that vertigo is prevalent in the elderly population, with estimates of incidence ranging from 20 to 58% (Lasisi and Gureje, 2014; Lindell et al., 2021; Fancello et al., 2023). The pathogenesis of vertigo is multifactorial and primarily characterized by illusions of rotational motion, often accompanied by symptoms such as nystagmus, postural imbalance, falls, and neurovegetative effects (Roque Reis et al., 2016; Du et al., 2022). These symptoms limit daily activities, significantly impacting the physical and mental health and overall quality of life of affected individuals.

The inner ear, with its complex metabolic mechanisms, can be adversely affected by alterations in blood concentrations of glucose and insulin, potentially leading to hearing loss and vestibular disorders (Albernaz, 2016). Approximately 20% of patients with dizziness also experience hearing loss (Sunitha et al., 2019). These patients often show severe cochlear damage and may have extensive or deep ischemia in the inner ear (Kuhn et al., 2011). Notably, the incidence of vertigo can reach 20–60% among individuals with sensorineural hearing loss (Rambold et al., 2005). The co-occurrence of sudden hearing loss (SHL) and vertigo, especially when occurring in close temporal proximity, has been associated with a higher risk of subsequent stroke compared to SHL or vertigo alone (Chang et al., 2018). This indicates that SHL in vertigo patients should not be viewed as merely a benign peripheral vestibular sign.

Given the potential severe consequences of concomitant hearing loss in elderly patients with vertigo and dizziness, this condition merits increased clinical attention. Accordingly, this study retrospectively analyzes hearing examination reports of elderly patients experiencing dizziness from outpatient hearing centers over a 10-year period. Our aim is to characterize the types of hearing loss in this specific population.

## 2. Materials and methods

### 2.1. Participants

This study involved retrospective collection and examination of outpatient diagnosis reports, pure tone audiometry, acoustic immittance measurement and auditory brainstem response (ABR) tests from January 2010 to December 2021. Participants were patients over 50 years old with vertigo and dizziness, visiting the General Hospital of the People's Liberation Army in the Chinese People's Republic. A total of 9,384 patients (3,582 males and 5,802 females, aged 50–96 years, average age 66.24 ± 7.04 years) with hearing loss were included. The study was approved by the PLA General Hospital's Ethics Committee (No. S2022-673-01), and all procedures complied with the 1964 Helsinki declaration and its later amendments or comparable ethical standards.

### 2.2. Hearing evaluation methods

#### 2.2.1. Pure tone audiometry

Post-routine otolaryngology examinations and history collection, the Astera pure tone audiometry (Natus, US) was used to obtain the hearing threshold. The EAR-3A insert phones were applied (American Speech-Language-Hearing Association, 2005), and frequencies from 250 Hz to 8 kHz were tested using the ascending method (ISO 8253-1: 2010).

#### 2.2.2. Tympanogram

The middle ear's tympanograms were obtained using TympStar clinical tympanometer (Grason-Stadler, US) and Titan tympanometer (Interacoustics, Denmark), with a probe tone of 226 Hz. Tympanograms were classified into five types (A, AD, AS, B, and C) according to the Liden-Jerger classification criteria.

#### 2.2.3. Auditory electrophysiology tests

ABR tests were conducted using the Eclipse EP25 platform (Interacoustics, Denmark) with insert earphones (3A, Etymotic Research, US). Alternating short-duration clicks with a repetition rate of 19.3 Hz were used as stimuli. Parameters for the test are detailed.

### 2.3. Pure-tone audiogram typing

**Figure 1:**
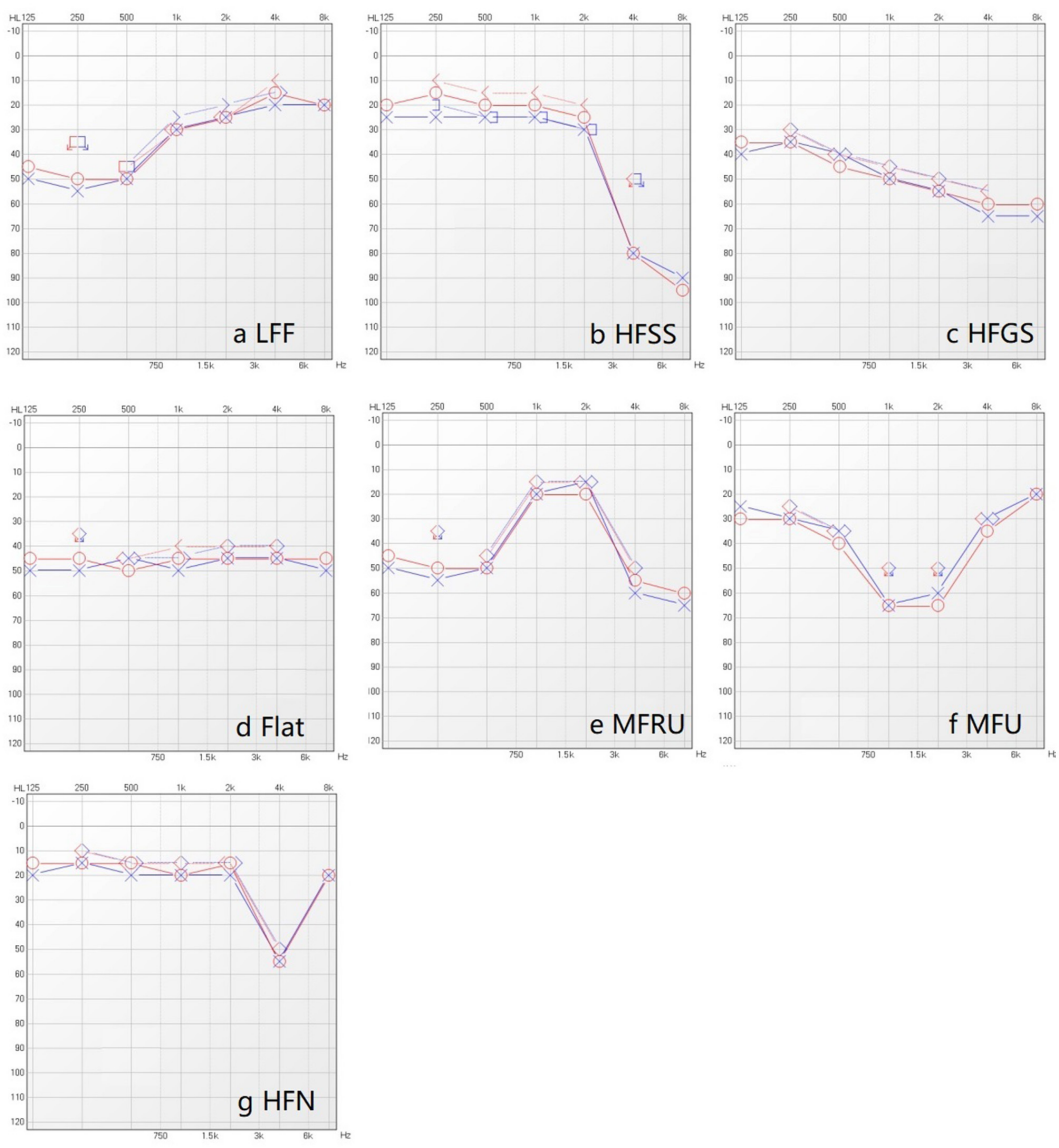
Seven typing of pure-tone audiogram. **(a)** Low frequency falling (LFF): The difference between the poor low frequency threshold (i.e., the larger listening threshold in 250 Hz and 500 Hz) and the good high frequency threshold (i.e., the smaller listening threshold in 4 kHz and 8 kHz) is greater than 15 dB, and the former is greater than the latter. **(b)** High frequency steeply sloping (HFSS): The difference between the mean value of 500 Hz and 1 kHz air conduction hearing thresholds and the average values of high frequency (4 kHz and 8 kHz) air conduction hearing thresholds is greater than 30 dB, and the former was smaller than the latter. **(c)** High frequency gently sloping (HFGS): The difference between the average values of air conduction threshold (500 Hz and 1 kHz) and the average value of high frequency air conduction thresholds (4 kHz and 8 kHz) is greater than 15 dB, meanwhile less than or equal to 29 dB, and the former is less than the latter. **(d)** Flat: The difference between the average values of the three air conduction hearing thresholds(low-frequency, medium-frequency, high-frequency) is less than 15 dB. **(e)** Mid frequency Reverse U-shape (MFRU): The difference between the medium frequency optimal threshold (i.e., the smaller threshold in 1 kHz and 2 kHz), the low frequency optimal threshold (i.e., the smaller threshold in 250 Hz and 500 Hz) and the high frequency optimal threshold (i.e., the smaller threshold in 4 kHz and 8 kHz) is more than 15 dB, and the medium frequency is better than (the threshold is less than) the low frequency and high frequency. **(f)** Mid frequency U-shape (MFU): The difference between the worst listening threshold of medium frequency (the larger threshold of 1 kHz and 2 kHz), the poor listening threshold of low frequency (the larger threshold of 250 Hz and 500 Hz) and the poor threshold of high frequency (the larger listening threshold of 4 kHz and 8 kHz) is more than 15 dB, and the medium frequency less than (the threshold is greater than) low frequency and high frequency. **(g)** High frequency notching (HFN): The threshold of 4 kHz is the worst, and the difference between 4kHz and other frequency thresholds is greater than 15 dB.

To facilitate the diagnostic classification of hearing loss in elderly patients, we adopted a typing criterion for pure-tone audiograms based on clinical observations and a review of existing literature (see Figure 1) (Demeester et al., 2009; Lee et al., 2020). We defined 250 and 500 Hz as “low frequency (LF),” 1 and 2 kHz as “middle frequency (MF),” and 4 and 8 kHz as “high frequency (HF).”

### 2.4. Inclusion and exclusion criteria

#### 2.4.1. Inclusion criteria

(1) Age ≥ 50 years;
(2) Diagnosed by a careful interview and vestibular function results by an otologist, and another specialist reviewed the clinical notes to confirm the diagnosis. All diagnoses could be divided into benign recurrent vertigo (BRV) (van Leeuwen et al., 2022), MD (Monsell et al., 1995), vestibular neuropathy (VN, the diagnosis was based on the history of acute sustained vertigo or imbalance, positive spontaneous nystagmus or unilateral weakness >25% in vHIT or unilateral VOR gain loss combined with obvious catch-up saccades in vHIT and no additional central lesion signs) (Haeussler et al., 2022), BPPV (Kim et al., 2021), functional and psychiatric vertigo (PV) (Traschütz et al., 2021), vestibular migraine (VM) (García et al., 2021), bilateral vestibular hypofunction (BVH) (Lucieer et al., 2016), delayed endolymphatic hydrops (DEH) (Reynard et al., 2018), others [including vestibular paroxysmia (VP), acoustic neurinoma (AN, radiologically diagnosed and went through vHIT before surgery), traumatic vertigo (TV, diagnosed by imaging), Ramsay Hunt Syndrome (RHS, diagnosed with an ipsilateral herpetic eruption on the auricle and external ear canal, facial palsy, and vertigo) and vascular vertigo, cervicogenic vertigo, tinnitus with vertigo, and complication with MD and VM, complication with MD and BPPV].
(3) The complete binaural (L, R) air conduction (125, 250, 500, 1,000, 2,000, 4,000, 8,000 Hz) and bone conduction (250, 500 Hz, 1, 2, 4 kHz) based on the data of pure-tone threshold;
(4) The results of acoustic immittance test and auditory brainstem response (ABR) test.

#### 2.4.2. Exclusion criteria

Participants were excluded from the study if they:

(1) Had incomplete data from the pure tone audiometry (either not done or if the air bone conduction threshold data was incomplete).
(2) Had missing age, gender, or diagnostic information.

### 2.5. Statistical analysis

In this study, the descriptive analysis was mainly used. The counting data was expressed as frequency (percentage). Comparison of pure tone hearing threshold and acoustic immittance measurement applied one-way ANOVA (When *P* < 0.05, there is a statistical difference). Least-significant difference (LSD) was used for *Post-hoc* Multiple Comparisons.

K-Means clustering was used for the secondary classification of audiograms. We extracted the features of the left and right audiogram curves, including maximum, minimum, mean, variance, slope of each inflection point, and curve distance as the feature values. These variables were standardized to ensure comparability, and the resulting dataset was used as the input for the subsequent K-means clustering. The optimal number of clusters was determined by fitting the K-means unsupervised machine learning algorithm to 3–6 clusters, respectively.

## 3. Results

### 3.1. Data overview

**Figure 2:**
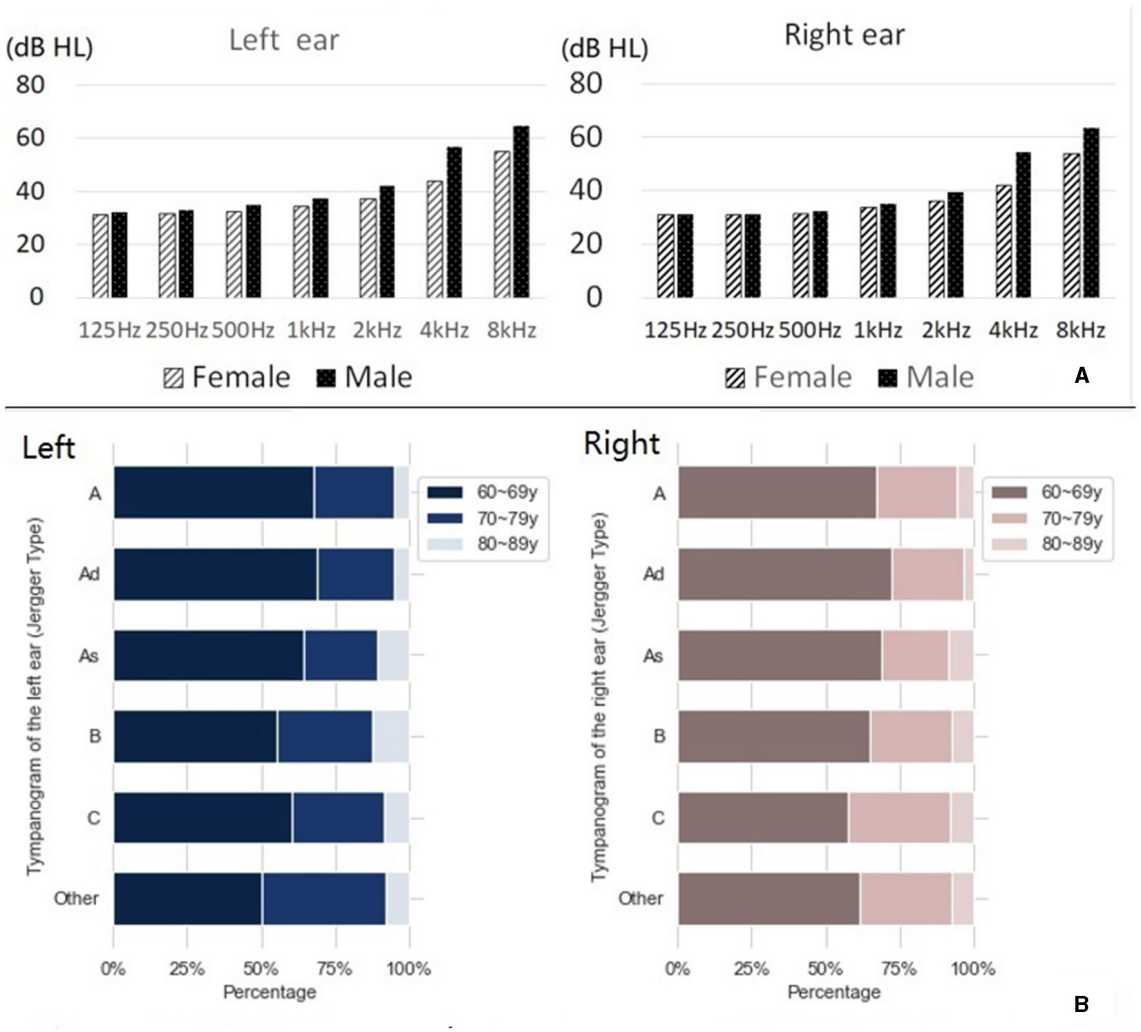
Hearing results of elderly patients with vertigo and dizziness. **(A)** Hearing thresholds for males and females at each frequency. **(B)** Jerger classification of tympanic diagram.

As depicted in Table 1, the largest group of patients with vertigo and dizziness was aged between 60 and 69 years old, with a total of 5,726 cases, which accounted for 61.02% of all admissions. There was a higher prevalence of female patients (61.83%) than male patients (38.17%). Male patients generally had a worse hearing threshold than females, with a difference of 10 dB HL observable at 4 k and 8 kHz (see Figure 2A). In the 2021 WHO's hearing classification, the largest category of hearing loss patients, comprising 44.63% (*n* = 4,188), was classified as “mild.”

**Table 1:** Basic information for patients with vertigo and dizziness.

**Variable**		**Number**	**Percentage**
Age (years)	50~59	1,108	11.81
	60~69	5,726	61.02
	70~79	2,072	22.08
	80~89	464	4.94
	90~99	14	0.15
Gender	Male	3,582	38.17
	Female	5,802	61.83
1997 Classification of hearing loss	0	4,166	44.39
	1	3,155	33.62
	2	1,580	16.84
	3	409	4.36
	4	74	0.79
2021WHO classification for hearing loss	Normal	1,701	18.13
	Mild	4,188	44.63
	Moderate	1,960	20.89
	Moderate to severe	777	8.28
	Severe	271	2.89
	Profond	62	0.66
	Total deafness	20	0.21
	Single sided deafness	405	4.32

### 3.2. Jerger classification of the tympanic diagram

Figure 2B shows the Jerger classification of tympanograms in patients with vertigo and dizziness (additional details can be found in Supplementary Table 2). There was a near equal distribution between left and right ears, with type A being the most common Jerger classification of tympanogram.

### 3.3. ABR results

Each wave latency and inter-wave period fell within the normal range, as shown in Table 2.

**Table 2:** ABR test results of patients with vertigo and dizziness.

**Test ear**	**ABR wave**	**Statistical description**	**60~69 (y)**	**70~79 (y)**	**80~89 (y)**
Left	I	Observed cases (unobserved cases)	162 (46)	51 (19)	4 (6)
		Mean ± SD	1.59 ± 0.20	1.57 ± 0.18	1.57 ± 0.21
		Median	1.57 (1.48~1.70)	1.52 (1.45~1.70)	1.61 (1.43~1.70)
Left	III	Observed cases (unobserved cases)	153 (55)	53 (17)	4 (6)
		Mean ± SD	3.86 ± 0.27	3.86 ± 0.26	3.91 ± 0.21
		Median	3.85 (3.70~4.00)	3.88 (3.70~4.03)	3.94 (3.77~4.06)
Left	V	Observed cases (unobserved cases)	194 (14)	70 (0)	8 (2)
		Mean ± SD	5.83 ± 0.38	5.84 ± 0.32	5.95 ± 0.32
		Median	5.73 (5.60~5.95)	5.85 (5.60~6.03)	5.85 (5.71~6.27)
Left	I-III	Observed cases (unobserved cases)	139 (69)	46 (24)	3 (7)
		Mean ± SD	2.27 ± 0.21	2.25 ± 0.17	2.46 ± 0.34
		Median	2.27 (2.15~2.35)	2.21 (2.13~2.33)	2.32 (2.21~2.85)
Left	III-V	Observed cases (unobserved cases)	153 (55)	53 (17)	4 (6)
		Mean ± SD	1.90 ± 0.16	1.92 ± 0.18	1.89 ± 0.12
		median	1.92 (1.80~2.00)	1.90 (1.82~2.00)	1.91 (1.81~1.97)
Left	I-V	Observed cases (unobserved cases)	162 (46)	51 (19)	4 (6)
		Mean ± SD	4.16 ± 0.21	4.18 ± 0.22	4.28 ± 0.41
		Median	4.14 (4.03~4.27)	4.15 (4.04~4.32)	4.16 (4.01~4.55)
Right	I	Observed cases (unobserved cases)	172 (36)	55 (15)	5 (5)
		Mean ± SD	1.59 ± 0.19	1.57 ± 0.16	1.70 ± 0.08
		Median	1.57 (1.48~1.69)	1.55 (1.48~1.68)	1.68 (1.63~1.73)
Right	III	Observed cases (unobserved cases)	165 (43)	54 (16)	6 (4)
		Mean ± SD	3.82 ± 0.22	3.85 ± 0.21	4.02 ± 0.27
		Median	3.81 (3.65~3.98)	3.85 (3.70~4.00)	3.91 (3.85~4.08)
Right	V	Observed cases (unobserved cases)	199 (9)	66 (4)	9 (1)
		Mean ± SD	5.80 ± 0.36	5.78 ± 0.27	5.95 ± 0.34
		Median	5.75 (5.58~5.95)	5.75 (5.63~5.92)	5.95 (5.70~6.22)
Right	I-III	Observed cases (unobserved cases)	149 (59)	47 (23)	4 (6)
		Mean ± SD	2.23 ± 0.18	2.25 ± 0.14	2.21 ± 0.11
		Median	2.23 (2.12~2.35)	2.23 (2.15~2.32)	2.20 (2.13~2.30)
Right	III-V	Observed cases (unobserved cases)	165 (43)	54 (16)	6 (4)
		Mean ± SD	1.91 ± 0.15	1.92 ± 0.17	1.93 ± 0.24
		Median	1.90 (1.82~2.00)	1.90 (1.83~2.00)	1.86 (1.80~2.12)
Right	I-V	Observed cases (unobserved cases)	172 (36)	55 (15)	5 (5)
		Mean ± SD	4.15 ± 0.20	4.17 ± 0.21	4.10 ± 0.25
		Median	4.13 (4.02~4.26)	4.15 (4.05~4.32)	4.15 (3.90~4.27)

### 3.4. Classification of audiogram shapes in elderly patients with vertigo and dizziness

**Figure 3:**
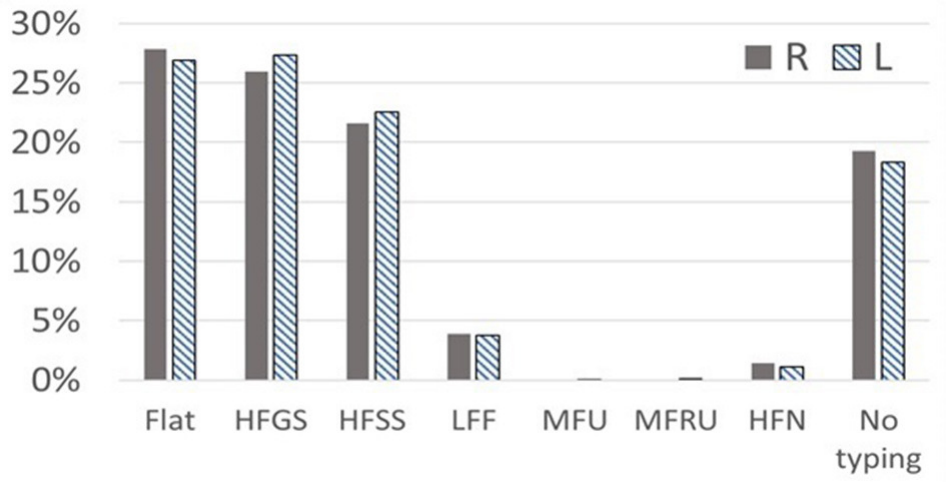
The classification of audiogram shapes in elderly patients with vertigo syndrome. Typing of pure-tone audiogram: Flat, HFGS (high frequency gently sloping), HFSS (high frequency steeply sloping), LFF (low frequency falling), MFU (mid frequency U-shape), MFRU (mid frequency Reverse U-shape), and HFN (high frequency notching).

Table 3 outlines the pure-tone audiogram typing and the corresponding proportions of patients with vertigo and dizziness. Similar patterns were observed in both left and right ears. Predominant audiometric shapes included flat (27.81% in the right ear, 26.89% in the left), high-frequency gently sloping (HFGS) (25.97% in the right ear, 27.34% in the left), and high-frequency steeply sloping (HFSS) (21.60% in the right ear, 22.53% in the left) (see Figure 3).

**Table 3:** Audiogram classification of patients with vertigo and dizziness.

**Audiogram classification**	**2010 (%)**	**2011 (%)**	**2012 (%)**	**2013 (%)**	**2014 (%)**	**2015 (%)**	**2016 (%)**	**2017 (%)**	**2018 (%)**	**2019 (%)**	**2020 (%)**	**2021 (%)**
Flat (R)	11 (26.19)	8 (32.00)	28 (20.14)	172 (26.88)	113 (25.11)	274 (27.45)	281 (25.57)	382 (27.21)	332 (25.62)	162 (29.24)	362 (34.77)	559 (32.96)
Flat (L)	11 (26.19)	5 (20.00)	29 (20.86)	153 (23.91)	109 (24.22)	250 (25.05)	260 (23.66)	367 (26.14)	345 (26.62)	160 (28.88)	353 (33.91)	545 (32.13)
HFGS (R)	7 (16.67)	7 (28.00)	42 (30.22)	164 (25.63)	118 (26.22)	295 (29.56)	324 (29.48)	352 (25.07)	369 (28.47)	146 (26.35)	244 (23.44)	438 (25.83)
HFGS (L)	12 (28.57)	8 (32.00)	41 (29.50)	183 (28.59)	138 (30.67)	290 (29.06)	343 (31.21)	390 (27.78)	388 (29.94)	136 (24.55)	264 (25.36)	437 (25.77)
HFSS (R)	11 (26.19)	5 (20.00)	35 (25.18)	166 (25.94)	108 (24.00)	225 (22.55)	250 (22.75)	332 (23.65)	278 (21.45)	125 (22.56)	194 (18.64)	355 (20.93)
HFSS (L)	14 (33.33)	7 (28.00)	37 (26.62)	174 (27.19)	98 (21.78)	233 (23.35)	275 (25.02)	356 (25.36)	293 (22.61)	136 (24.55)	199 (19.12)	345 (20.34)
LFF (R)	2 (4.76)	0 (0.00)	7 (5.04)	19 (2.97)	16 (3.56)	25 (2.51)	37 (3.37)	53 (3.77)	72 (5.56)	15 (2.71)	50 (4.80)	79 (4.66)
LFF (L)	0 (0.00)	1 (4.00)	7 (5.04)	13 (2.03)	10 (2.22)	27 (2.71)	27 (2.46)	50 (3.56)	73 (5.63)	19 (3.43)	51 (4.90)	86 (5.07)
MFU (R)	0 (0.00)	0 (0.00)	0 (0.00)	0 (0.00)	0 (0.00)	0 (0.00)	0 (0.00)	0 (0.00)	1 (0.08)	0 (0.00)	1 (0.10)	0 (0.00)
MFU (L)	0 (0.00)	0 (0.00)	0 (0.00)	0 (0.00)	0 (0.00)	0 (0.00)	0 (0.00)	0 (0.00)	0 (0.00)	0 (0.00)	1 (0.10)	0 (0.00)
MFRU (R)	0 (0.00)	0 (0.00)	0 (0.00)	0 (0.00)	3 (0.67)	0 (0.00)	0 (0.00)	0 (0.00)	1 (0.08)	0 (0.00)	0 (0.00)	2 (0.12)
MFRU (L)	0 (0.00)	0 (0.00)	1 (0.72)	2 (0.31)	1 (0.22)	0 (0.00)	0 (0.00)	1 (0.07)	0 (0.00)	0 (0.00)	1 (0.10)	1 (0.06)
HFN (R)	0 (0.00)	0 (0.00)	1 (0.72)	8 (1.25)	5 (1.11)	15 (1.50)	16 (1.46)	23 (1.64)	15 (1.16)	4 (0.72)	17 (1.63)	31 (1.83)
HFN (L)	0 (0.00)	1 (4.00)	1 (0.72)	6 (0.94)	3 (0.67)	11 (1.10)	9 (0.82)	10 (0.71)	13 (1.00)	6 (1.08)	14 (1.34)	30 (1.77)
No typing (R)	12 (28.57)	5 (20.00)	29 (20.86)	128 (20.00)	101 (22.44)	190 (19.04)	217 (19.75)	299 (21.30)	267 (20.60)	111 (20.04)	208 (19.98)	291 (17.16)
No typing (L)	6 (14.29)	4 (16.00)	27 (19.42)	121 (18.91)	96 (21.33)	206 (20.64)	206 (18.74)	258 (18.38)	227 (17.52)	108 (19.49)	188 (18.06)	313 (18.46)

HFGS, High frequency gently sloping; HFSS, High frequency steeply sloping; LFF, Low frequency falling; MFU, Mid frequency U-shape; MFRU, Mid frequency Reverse U-shape; HFN, High frequency notching.

However, Figure 3 also reveals a significant number of patients categorized as “No typing” (18.3% in the left ear, 19.25% in the right) based on existing criteria, suggesting that current hearing classification standards may not be entirely suitable for elderly vertigo and dizziness patients with hearing loss.

Upon reclassification of the pure tone results (*n* = 9,299), K-Means clustering analysis suggested that the optimal number of clusters was three (Table 4). The ANOVA statistical results of each characteristic value showed *P* = 0.000, with sample sizes for the three clusters being 2,747, 2,413, and 4,139, respectively.

**Table 4:** K-Means clustering analysis results.

**Cluster**	**Sample sizes**					
3	1	2	3			
	2,747	2,413	4,139			
4	1	2	3	4		
	4,147	1	2,745	2,406		
5	1	2	3	4	5	
	2,618	1,160	1,633	3,887	1	
6	1	2	3	4	5	6
	1,505	1	2,171	3,030	1,107	1,485

### 3.5. Auditory examination results of patients with definite diagnosis

**Figure 4:**
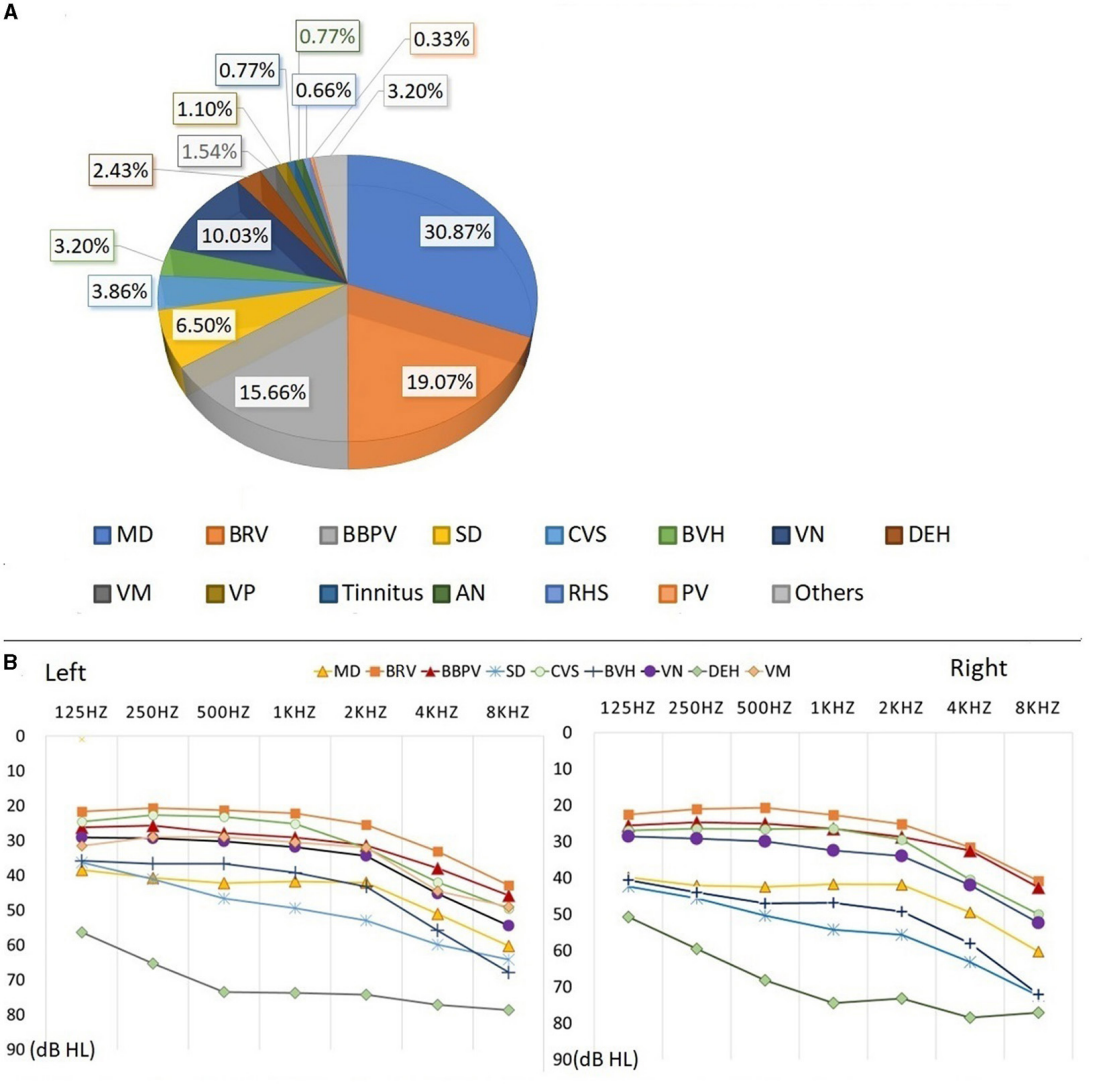
Auditory examination results of patients with definite diagnosis. The percentage of different vestibular syndrome is shown in this figure [Diagnosis types of vestibular syndrome: Meniere disease (MD), Benign recurrent vertigo (BRV), Benign paroxysmal positional vertigo (BPPV), Sudden deafness (SD), Chronic vestibular syndrome (CVS), Bilateral vestibular hypofunction (BVH), Vestibular neuropathy (VN), Delayed endolymphatic hydrops (DEH) Vestibular migraine (VM), Vestibular paroxysmia (VP), Tinnitus, Acoustic neurinoma (AN), Ramsay Hunt Syndrome (RHS), Psychiatric vertigo (PV)]. Eight diseases (MD, BRV, BBPV, VN, SD, CVS, BVH, and DEH) with a higher proportion are analyzed. The percentage of remaining diseases(VM, VP, Tinnitus, AN, RHS, PV and other diseases) is less than 2.00%. **(A)** Hearing thresholds of MD, BRV, BBPV, VN, SD, CVS, BVH, and DEH are shown in this figure. **(B)** The patient's hearing threshold gradually increases from 125 to 8,000 Hz. The hearing threshold of 8000 Hz can be 17.03–32.00dB HL higher than that of 125 Hz. The average hearing thresholds (500, 1,000, 2,000, 4,000 Hz) for eight types of vestibular diseases were calculated, and the results show that he hearing threshold of DEH is higher than other vestibular diseases, at around 75 dB HL. The average hearing threshold of other seven vestibular diseases is between 25.04 and 55.86 dB HL.

Among the 907 elderly patients with definitively diagnosed vestibular syndrome in this study, the three most prevalent were Ménière's disease (MD, 30.87%), benign recurrent vertigo (BRV, 19.07%), and benign paroxysmal positional vertigo (BBPV, 15.66%). The distribution of these diseases is presented in Figure 4A.

Auditory examination results from the eight main types of vestibular syndrome (each comprising more than 2.00% of the total) were analyzed, with the corresponding auditory thresholds depicted in Figure 4B. The hearing thresholds in low, medium, and high frequencies for the left and right ears are presented in Table 5. Statistical analysis revealed significant differences in hearing thresholds among the various vestibular diseases (*P* < 0.001). Detailed results from multiple comparisons of hearing thresholds for different diseases are provided in Table 6.

**Table 5:** Mean and standard deviation of low, medium and high frequency hearing threshold for different vestibular diseases in the left and right ears.

**Type**	**LLF**	**LMF**	**LHF**	**RLF**	**RMF**	**RHF**
MD	39.56 ± 21.97	42.00 ± 23.76	55.61 ± 23.51	40.99 ± 21.68	42.00 ± 23.98	54.94 ± 24.44
BRV	21.10 ± 10.99	22.95 ± 12.52	38.02 ± 22.07	21.76 ± 10.29	22.85 ± 11.42	36.22 ± 21.04
BPPV	25.93 ± 19.80	29.41 ± 23.96	41.81 ± 24.73	25.16 ± 16.21	26.76 ± 19.50	37.54 ± 22.98
VN	29.22 ± 18.53	32.12 ± 21.40	49.78 ± 24.95	28.84 ± 19.43	32.13 ± 25.75	47.16 ± 26.70
SD	38.70 ± 27.32	49.66 ± 34.68	61.99 ± 32.62	43.94 ± 28.70	53.43 ± 34.45	67.78 ± 35.74
CVS	23.53 ± 16.17	26.77 ± 15.81	45.67 ± 23.85	26.64 ± 18.19	27.47 ± 17.80	45.24 ± 25.51
BVH	36.20 ± 17.40	39.67 ± 21.05	61.80 ± 24.75	42.30 ± 21.11	47.67 ± 27.03	65.00 ± 27.59
DEH	60.79 ± 27.78	73.77 ± 37.70	77.89 ± 35.38	33.55 ± 22.50	41.05 ± 35.26	53.16 ± 33.66
F	21.509	22.468	14.972	20.674	18.868	16.792
*P*	0.000	0.000	0.000	0.000	0.000	0.000

**Table 6:** Multiple comparison results of different vestibular diseases in the left and right ears.

**LLF**	**MD**	**BRV**	**BPPV**	**VN**	**SD**	**CVS**	**BVH**	**DEH**
MD		0.000	0.000	0.000	0.771	0.000	0.415	0.000
BRV	0.000		0.049	0.003	0.000	0.539	0.000	0.000
BPPV	0.000	0.049		0.256	0.000	0.560	0.019	0.000
VN	0.000	0.003	0.256		0.006	0.183	0.599	0.000
SD	0.771	0.000	0.000	0.006		0.001	0.599	0.000
CVS	0.000	0.539	0.560	0.001	0.001		0.019	0.000
BVH	0.415	0.000	0.019	0.599	0.599	0.019		0.000
DEH	0.000	0.000	0.000	0.000	0.000	0.000	0.000	
**LMF**	**MD**	**BRV**	**BPPV**	**VN**	**SD**	**CVS**	**BVH**	**DEH**
MD		0.000	0.000	0.001	0.025	0.001	0.625	0.000
BRV	0.000		0.023	0.003	0.000	0.407	0.001	0.000
BPPV	0.000	0.023		0.417	0.000	0.579	0.042	0.000
VN	0.001	0.003	0.417		0.000	0.278	0.149	0.000
SD	0.025	0.000	0.000	0.000		0.000	0.070	0.000
CVS	0.001	0.407	0.579	0.278	0.000		0.038	0.000
BVH	0.625	0.001	0.042	0.149	0.070	0.038		0.000
DEH	0.000	0.000	0.000	0.000	0.000	0.000	0.000	
**LHF**	**MD**	**BRV**	**BPPV**	**VN**	**SD**	**CVS**	**BVH**	**DEH**
MD		0.000	0.000	0.067	0.086	0.041	0.234	0.000
BRV	0.000		0.219	0.001	0.000	0.127	0.000	0.000
BPPV	0.000	0.219		0.029	0.000	0.456	0.000	0.000
VN	0.067	0.001	0.029		0.005	0.444	0.034	0.000
SD	0.086	0.000	0.000	0.005		0.004	0.975	0.016
CVS	0.041	0.127	0.456	0.444	0.004		0.017	0.000
BVH	0.234	0.000	0.000	0.034	0.975	0.017		0.033
DEH	0.000	0.000	0.000	0.000	0.016	0.000	0.033	
**RLF**	**MD**	**BRV**	**BPPV**	**VN**	**SD**	**CVS**	**BVH**	**DEH**
MD		0.000	0.000	0.000	0.307	0.000	0.745	0.105
BRV	0.000		0.157	0.008	0.000	0.211	0.000	0.012
BPPV	0.000	0.157		0.193	0.000	0.713	0.000	0.080
VN	0.000	0.008	0.193		0.000	0.597	0.002	0.338
SD	0.307	0.000	0.000	0.000		0.000	0.726	0.043
CVS	0.000	0.211	0.713	0.597	0.000		0.003	0.224
BVH	0.745	0.000	0.000	0.002	0.726	0.003		0.136
DEH	0.105	0.012	0.080	0.338	0.043	0.224	0.136	
**RMF**	**MD**	**BRV**	**BPPV**	**VN**	**SD**	**CVS**	**BVH**	**DEH**
MD		0.000	0.000	0.001	0.025	0.001	0.625	0.000
BRV	0.000		0.023	0.003	0.000	0.407	0.001	0.000
BPPV	0.000	0.023		0.417	0.000	0.579	0.042	0.000
VN	0.001	0.003	0.417		0.000	0.278	0.149	0.000
SD	0.025	0.000	0.000	0.000		0.000	0.070	0.000
CVS	0.001	0.407	0.579	0.278	0.000		0.038	0.000
BVH	0.625	0.001	0.042	0.149	0.070	0.038		0.000
DEH	0.000	0.000	0.000	0.000	0.000	0.000	0.000	
**RHF**	**MD**	**BRV**	**BPPV**	**VN**	**SD**	**CVS**	**BVH**	**DEH**
MD		0.000	0.000	0.017	0.001	0.050	0.058	0.766
BRV	0.000		0.675	0.002	0.000	0.078	0.000	0.006
BPPV	0.000	0.675		0.010	0.000	0.144	0.000	0.013
VN	0.017	0.002	0.010		0.000	0.726	0.002	0.352
SD	0.001	0.000	0.000	0.000		0.000	0.649	0.030
CVS	0.050	0.078	0.144	0.726	0.000		0.004	0.288
BVH	0.058	0.000	0.000	0.002	0.649	0.004		0.124
DEH	0.766	0.006	0.013	0.352	0.030	0.288	0.124	

## 4. Discussion

From the overall result of elderly patients with vertigo and dizziness, 83.10% of them were aged from 60 to 79. Among them, the grading of hearing loss was mainly in level 1 (44.63%) and 2(20.89%), indicating that elderly patients with vertigo and dizziness generally have mild to moderate hearing loss in this age range. Previous studies abroad have shown that caloric test responses depend on several factors that could be affected by age, such as ear canal volume, temporal bone thickness, and blood supply to the temporal bone (Enrietto et al., 1999). Several studies have found that caloric responses tend to increase in middle age with a peak between 50 and 70 years, and then decline modestly thereafter (Fernández et al., 2015). In clinical diagnosis, it's difficult to obtain a complete, meaningful, and treatment-oriented diagnosis in elderly dizzy patients. More than half of elderly patients with balance disorders are vague, inconsistent, or contradictory in describing their symptoms (Newman-Toker et al., 2007). Besides, there is not a single symptom that can predict with specificity the underlying causes of dizziness, and most of the time, elderly patients have more than one cause of dizziness (Fernández et al., 2015). In this study, the incidence of dizziness in females is higher than that in males, but the hearing threshold of females is better.

In this study, the tympanogram of elderly vertigo patients was mainly classified as type A. The wave latency and inter wave period of ABR were within normal range. Analyzing the cause of deafness may be related to blood supply disorders in the inner ear. According to the theory of internal ear blood supply disorder, the labyrinthine artery is the main artery of internal ear blood supply. When the labyrinthine artery has thrombosis, embolism or vasospasm, it will cause labyrinthine artery blood supply disorder, leading to sudden deafness; At the same time, since the labyrinthine artery enters the inner ear and is divided into the common cochlear artery and the vestibular artery, when the blood supply of the labyrinthine artery is impaired, the vestibular function of the patient will also be affected, and vertigo symptoms will appear (Prince and Stucken, 2021). The appearance of vestibular symptoms such as dizziness indicates the severity of the disease and the breadth of the lesion. In previous studies, the wave latency and inter wave period of ABR in elderly patients should be prolonged (Gupta et al., 2014). This phenomenon did not occur in this study, which may be related to the low patient sample size in the age group over 80 years old. For elderly people, it is also necessary to give a special normal reference value for the judgment of each wave latency and wave interval.

In this study, the main audiometric shapes of elderly patients with dizziness were flat, high-frequency gently sloping (HFGS) and high-frequency steeply sloping (HFSS). Due to the large oxygen consumption of the cochlea bottom, the metabolic rate is high. Compared with the cochlea top, the blood supply of the cochlea bottom is poor, and its auditory hair cell is more vulnerable to damage. The cause of deafness in patients with dizziness involves a wide range of surrounding organs, affecting the vestibular area. Moreover, as a result of the bottom of the cochlea near the vestibule, patients with dizziness may have relatively severe cochlear damage, and their inner ear may have a larger or deeper degree of ischemia (Yu and Li, 2018). From Figure 3, it can be seen that a large number of deaf patients are classified as having “No typing” (18.3% in the left ear, and 19.25% in the right ear) based on the current criteria. Presbycusis patients with vertigo and dizziness are often associated with complicated basic diseases such as diabetes, hypertension and coronary heart disease, and the degree of hearing loss is high and cause the diversity of hearing changes in elderly deaf patients. This indicated the possibility of inappropriate classification methods for elderly patients with hearing loss according to the fixed criteria for audiometric classification. Further detailed research is needed to analyze the hearing status of aged patients with different diseases.

## 5. Conclusion

Our study revealed that elderly patients with vertigo and dizziness primarily experienced mild to moderate hearing loss. Interestingly, we found that the hearing threshold of female patients was generally better than that of their male counterparts. We also discovered that the most common audiometric shapes in these patients were flat, high-frequency gently sloping (HFGS), and high-frequency steeply sloping (HFSS). Importantly, we identified significant differences in hearing thresholds across various vestibular diseases.

## Data availability statement

The original contributions presented in the study are included in the article/Supplementary material, further inquiries can be directed to the corresponding author.

## Ethics statement

The studies involving humans were approved by PLA General Hospital's Ethics Committee (No. S2022-673-01). The studies were conducted in accordance with the local legislation and institutional requirements. The participants provided their written informed consent to participate in this study. Written informed consent was obtained from the individual(s) for the publication of any potentially identifiable images or data included in this article.

## Author contributions

QW performed the methodology and writing—original draft. AC performed the data curation. MH performed the formal analysis. XL performed the writing—review and editing. YD performed the visualization. ZW performed the supervision. FJ performed the conceptualization, project administration, and funding acquisition. All authors contributed to the article and approved the submitted version.
