# Supplementary material for: Investigation of hearing loss in elderly vertigo and dizziness patients in the past 10 years
Source: Front Aging Neurosci. 2023 Sep 15;15:1225786. doi: 10.3389/fnagi.2023.1225786 (PMC10543661; doi:10.3389/fnagi.2023.1225786)
Supplement: Supplementary file 1 [file Table_1.DOCX]

Supplementary Materials for Investigation of Hearing Loss in Elderly Vertigo and Dizziness Patients in the Past Ten Years

**Table S1 Basic information of patients with vertigo and dizziness over the past 10 years**

| **Variable** | | **2010(%)** | **2011(%)** | **2012(%)** | **2013(%)** | **2014(%)** | **2015(%)** | **2016(%)** | **2017(%)** | **2018(%)** | **2019(%)** | **2020(%)** | **2021(%)** |
| --- | --- | --- | --- | --- | --- | --- | --- | --- | --- | --- | --- | --- | --- |
|  |  | **N=42** | **N=25** | **N=139** | **N=640** | **N=450** | **N=998** | **N=1099** | **N=1404** | **N=1296** | **N=554** | **N=1041** | **N=1696** |
| Age | |  |  |  |  |  |  |  |  |  |  |  |  |
|  | 50~59y | 0(0.00) | 0(0.00) | 0(0.00) | 0(0.00) | 0(0.00) | 0(0.00) | 0(0.00) | 23(1.64) | 1(0.08) | 13(2.35) | 430(41.31) | 641(37.79) |
|  | 60~69y | 27(64.29) | 14(56.00) | 94(67.63) | 438(68.44) | 319(70.89) | 684(68.54) | 740(67.33) | 961(68.45) | 909(70.14) | 351(63.36) | 441(42.36) | 748(44.10) |
|  | 70~79y | 15(35.71) | 8(32.00) | 37(26.62) | 164(25.63) | 109(24.22) | 276(27.66) | 290(26.39) | 329(23.43) | 303(23.38) | 156(28.16) | 144(13.83) | 241(14.21) |
|  | 80~89y | 0(0.00) | 3(12.00) | 8(5.76) | 37(5.78) | 22(4.89) | 38(3.81) | 67(6.10) | 89(6.34) | 80(6.17) | 32(5.78) | 24(2.31) | 64(3.77) |
|  | 90~99y | 0(0.00) | 0(0.00) | 0(0.00) | 1(0.16) | 0(0.00) | 0(0.00) | 2(0.18) | 2(0.14) | 3(0.23) | 2(0.36) | 2(0.19) | 2(0.12) |
| Gender | |  |  |  |  |  |  |  |  |  |  |  |  |
|  | Male | 21(50.00) | 10(40.00) | 54(38.85) | 250(39.06) | 170(37.78) | 379(37.98) | 408(37.12) | 541(38.53) | 495(38.19) | 212(38.27) | 419(40.25) | 623(36.73) |
|  | Female | 21(50.00) | 15(60.00) | 85(61.15) | 390(60.94) | 280(62.22) | 619(62.02) | 691(62.88) | 863(61.47) | 801(61.81) | 342(61.73) | 622(59.75) | 1073(63.27) |
| 1997Classification of hearing loss | |  |  |  |  |  |  |  |  |  |  |  |  |
|  | 0 | 23(54.76) | 12(48.00) | 51(36.69) | 256(40.00) | 177(39.33) | 417(41.78) | 461(41.95) | 590(42.02) | 517(39.89) | 235(42.42) | 541(51.97) | 886(52.24) |
|  | 1 | 12(28.57) | 7(28.00) | 57(41.01) | 244(38.13) | 151(33.56) | 369(36.97) | 415(37.76) | 512(36.47) | 415(32.02) | 174(31.41) | 319(30.64) | 480(28.30) |
|  | 2 | 7(16.67) | 4(16.00) | 25(17.99) | 113(17.66) | 96(21.33) | 156(15.63) | 180(16.38) | 228(16.24) | 273(21.06) | 117(21.12) | 138(13.26) | 243(14.33) |
|  | 3 | 0(0.00) | 2(8.00) | 5(3.60) | 26(4.06) | 24(5.33) | 47(4.71) | 37(3.37) | 61(4.34) | 80(6.17) | 27(4.87) | 35(3.36) | 65(3.83) |
|  | 4 | 0(0.00) | 0(0.00) | 1(0.72) | 1(0.16) | 2(0.44) | 9(0.90) | 6(0.55) | 13(0.93) | 11(0.85) | 1(0.18) | 8(0.77) | 22(1.30) |
| WHO Classification of hearing loss | |  |  |  |  |  |  |  |  |  |  |  |  |
|  | Normal | 9(21.43) | 0(0.00) | 18(12.95) | 99(15.47) | 77(17.11) | 154(15.43) | 200(18.20) | 223(15.88) | 194(14.97) | 94(16.97) | 257(24.69) | 376(22.17) |
|  | Mild | 21(50.00) | 15(60.00) | 71(51.08) | 311(48.59) | 186(41.33) | 479(48.00) | 490(44.59) | 655(46.65) | 543(41.90) | 236(42.60) | 443(42.56) | 738(43.51) |
|  | Moderate | 8(19.05) | 3(12.00) | 31(22.30) | 137(21.41) | 105(23.33) | 216(21.64) | 262(23.84) | 311(22.15) | 309(23.84) | 123(22.20) | 176(16.91) | 279(16.45) |
|  | Moderate to severe | 2(4.76) | 1(4.00) | 10(7.19) | 57(8.91) | 49(10.89) | 85(8.52) | 83(7.55) | 104(7.41) | 137(10.57) | 64(11.55) | 57(5.48) | 128(7.55) |
|  | Severe | 0(0.00) | 2(8.00) | 4(2.88) | 10(1.56) | 13(2.89) | 26(2.61) | 21(1.91) | 43(3.06) | 57(4.40) | 17(3.07) | 27(2.59) | 51(3.01) |
|  | Profond | 0(0.00) | 0(0.00) | 1(0.72) | 2(0.31) | 1(0.22) | 7(0.70) | 5(0.45) | 13(0.93) | 7(0.54) | 3(0.54) | 5(0.48) | 18(1.06) |
|  | Total deafness | 0(0.00) | 0(0.00) | 0(0.00) | 1(0.16) | 2(0.44) | 2(0.20) | 1(0.09) | 2(0.14) | 5(0.39) | 0(0.00) | 3(0.29) | 4(0.24) |
|  | Single sided deafness | 2(4.76) | 4(16.00) | 4(2.88) | 23(3.59) | 17(3.78) | 29(2.91) | 37(3.37) | 53(3.77) | 44(3.40) | 17(3.07) | 73(7.01) | 102(6.01) |

| Test ear | Jerger typing | 60~69(y) | 70~79(y) | 80~89(y) | total |
| --- | --- | --- | --- | --- | --- |
| Right | A | 1565(66.88%) | 636(27.18%) | 138(5.90%) | 2340 |
|  | Ad | 218(72.43%) | 73(24.25%) | 10(3.32%) | 301 |
|  | As | 165(68.46%) | 54(22.41%) | 20(8.30%) | 241 |
|  | B | 35(63.64%) | 15(27.27%) | 4(7.27%) | 55 |
|  | C | 124(56.88%) | 74(33.94%) | 18(8.26%) | 218 |
|  | Others | 8(61.54%) | 4(30.77%) | 1(7.69%) | 13 |
| Left | A | 1615(67.71%) | 640(26.83%) | 127(5.32%) | 2385 |
|  | Ad | 198(68.75%) | 75(26.04%) | 15(5.21%) | 288 |
|  | As | 151(63.98%) | 59(25.00%) | 25(10.59%) | 236 |
|  | B | 31(55.36%) | 18(32.14%) | 7(12.50%) | 56 |
|  | C | 114(59.69%) | 59(30.89%) | 16(8.38%) | 191 |
|  | Others | 6(50.00%) | 5(41.67%) | 1(8.33%) | 12 |

**Table S2 Jerger classification of tympanogram in patients with vertigo and dizziness**
